# Seroprevalence and Potential Correlation of Transfusion-Transmitted Infections With the ABO Blood Group System Among Blood Donors in Eastern India

**DOI:** 10.7759/cureus.115870

**Published:** 2026-09-06

**Authors:** Nishith Nayan, Bankim Das, Rakesh Kumar, Saurabh Lahre, Sweta Ranjan

**Affiliations:** 1 Transfusion Medicine and Blood Bank, All India Institute of Medical Sciences, Patna, Patna, IND; 2 Transfusion Medicine and Blood Bank, Medanta Hospital, Noida, IND

**Keywords:** abo blood group system, blood donors, blood safety, blood transfusion, seroprevalence, transfusion transmitted infections (ttis)

## Abstract

Background: Transfusion-transmitted infections (TTIs) remain a significant concern in transfusion medicine due to their impact on blood safety. Understanding the distribution of TTIs among blood donors and exploring potential associations with the ABO blood group system can enhance donor screening strategies.

Materials and methods: This cross-sectional retrospective study was conducted among blood donors from January 2020 to December 2024. Blood donors were tested for ABO and RhD blood groups and for five mandatory TTI markers: human immunodeficiency virus (HIV), hepatitis B virus (HBV), hepatitis C virus (HCV), syphilis, and malaria. The chi-square test and odds ratio (OR) were used to determine the association of TTIs with ABO and RhD blood groups.

Results: Out of 65,160 healthy donors, 2167 (3.33%) were positive for various TTIs. HBV had the maximum prevalence (n = 1017 (1.56%)), followed by syphilis (n = 655 (1.01%)), HCV (n = 268 (0.41%)), and HIV (n = 188 (0.29%)). Coinfection was seen in 0.06% of donors (n = 39). The maximum number of seroreactive donors was found among blood group B (n = 776 (3.27%)), followed by group O (n = 719 (3.35%)), group A (n = 482 (3.38%)), and group AB (n = 190 (3.34%)). However, the overall association of TTIs with ABO and RhD blood groups was not statistically significant, except for HBsAg, which had a statistically significant association with blood group B.

Conclusion: Hepatitis B was the most prevalent transfusion-transmitted infection among blood donors, with a statistically significant association with blood group B. While no overall significant correlation was found between ABO or RhD blood groups and TTIs, the findings underscore the ongoing risk posed by hepatitis B. This highlights the need for sustained public health efforts, targeted awareness programs, and stringent donor screening protocols to enhance transfusion safety and reduce the TTI burden.

## Introduction

Transfusion-transmitted infections (TTIs) remain a major challenge to blood safety, particularly in regions with high transfusion demands such as Eastern India. Despite advances in screening strategies, TTIs continue to threaten transfusion practices, as they can result in significant morbidity and mortality among recipients. In India, mandatory donor screening includes human immunodeficiency virus (HIV), hepatitis B virus (HBV), hepatitis C virus (HCV), syphilis, and malaria [1].

Globally, the prevalence of TTIs among blood donors has been reported by the World Health Organization (WHO) to vary from 0.005 to 6.02% for HBV, 0.002-1.67% for HCV, 0.001-1.60% for HIV, and 0.02%-1.81% for syphilis [2]. In India, the prevalence of HBV is 0.45-1.57%, HCV 0.05-0.83%, HIV 0.05-0.18%, and syphilis 0.02-0.48% [3]. Multiple studies from across India have reported variable prevalence of TTIs among blood donors. Overall, TTI reactivity among blood donors in India shows marked regional heterogeneity, with reported ranges of 0.5-1.8% in Southern India, 0.7-2.2% in Western India, 0.8-2.4% in Northern India, and 1.0-3.1% in Eastern India, with HBV consistently identified as the most prevalent transfusion-transmissible infection [4-7]. These variations highlight both global and regional differences in disease burden, underscoring the importance of continuous epidemiological surveillance and evidence-based transfusion policies [8].

In addition to overall prevalence, researchers have explored potential associations between ABO blood groups and infection susceptibility. Several studies suggest that individuals with blood group A may have an increased risk for certain viral infections, while those with group O may have relatively lower susceptibility [9]. Pathogen interactions with specific blood group antigens may facilitate infection, although the associations appear to be pathogen-specific and population-dependent. The evidence for such susceptibility remains inconsistent and varies by population and pathogen. Importantly, most available data are from North and South India, while comprehensive data from Eastern India are limited. Given potential regional variations in blood group distribution, donor characteristics, and infection patterns, focused research is crucial to tailor donor screening strategies and transfusion safety protocols to the needs of the local population.

The present study was therefore undertaken to investigate the seroprevalence of TTIs and their possible correlation with ABO and RhD blood groups among blood donors in Eastern India. The findings aim to fill an existing knowledge gap and provide evidence that can guide targeted interventions, policy development, and improved transfusion safety in the region.

This work was previously presented as a meeting abstract at the TRANSCON 2025 Annual National Conference of the Indian Society of Blood Transfusion & Immunohaematology on September 20, 2025.

## Materials and methods

Study setting and design

This was a cross-sectional study conducted at the Department of Transfusion Medicine in a tertiary care hospital in Eastern India from January 2020 to December 2024. All healthy blood donors, both voluntary and replacement, were included in this study for analysis, in accordance with the guidelines for blood donor selection and deferral of the National Blood Transfusion Council (2025) [10]. They were screened for five mandatory TTIs (HIV, HBV, HCV, syphilis, and malaria) as per the guidelines laid down by the Drugs and Cosmetics (D&C) Act, 1940, and Rules, 1945, Government of India [11]. Methods employed for blood grouping and TTI testing were as per the departmental standard operating procedure. Donor samples were collected as per the blood donation protocol in plain and ethylenediaminetetraacetic acid (EDTA) vials from the sample pouch at the time of donation for testing. No extra samples were collected from any other venipuncture site. Malaria was not included in the study because our blood center had not encountered any positive cases over the past three years.

Study participants

Inclusion Criteria

Donors who met the eligibility criteria set by the Drugs and Cosmetics Act, 1940, NBTC guidelines (2025), and departmental standard operating procedure (SOP) were included in the study [10].

Exclusion Criteria

Donors who were deferred from blood donation according to the guidelines and SOP or who did not consent to participation in the study were excluded [10].

The whole study protocol is shown in Figure 1.

**Figure 1:**
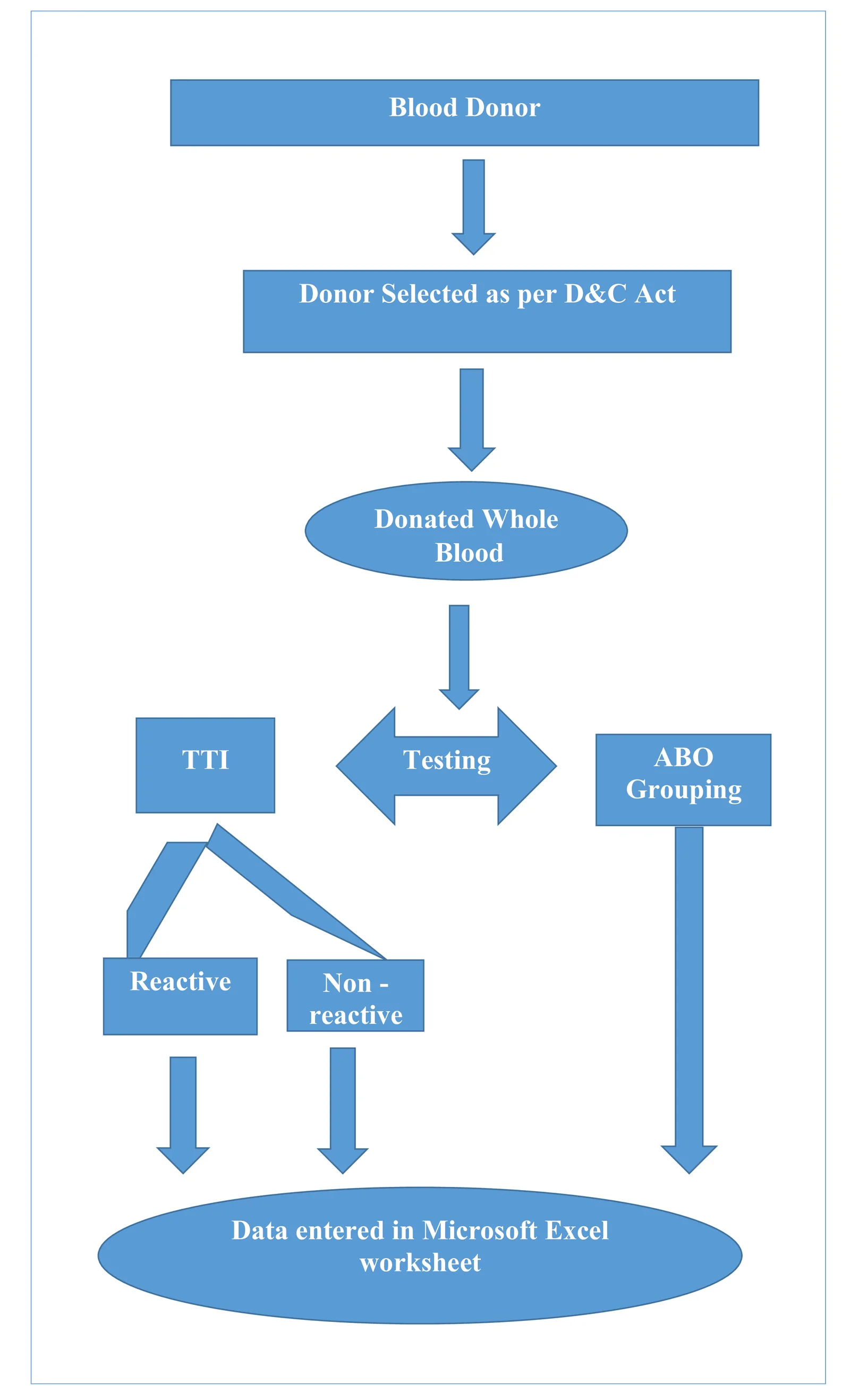
Study protocol. TTI: transfusion-transmitted infection; ABO: ABO blood group system.

Test methodology

Blood Grouping

Both forward and reverse blood grouping were performed by the column agglutination technique (CAT) using a blood grouping gel card (BIO-RAD ID-Card DiaClon ABO/D + Reverse Grouping, United States) in strict accordance with the manufacturer’s instructions [12].

Testing for TTIs

A chemiluminescent immunoassay (CLIA) was performed using the VITROS ECiQ 3600 (QuidelOrtho™, Raritan, New Jersey) according to the manufacturer’s instructions. The assay is designed for the qualitative detection of markers for TTIs, including HIV, HBV, HCV, and syphilis.

Ethical approval

The study received approval from the Institutional Review Board (IRB) and the Institutional Ethics Committee (IEC) (RD/AIIMS/Pat/2025/RAC/106).

Statistical analysis

Statistical analysis was performed by entering the data (ABO group and RhD typing, sex, age, and TTI seroprevalence for HBV, HCV, HIV, and syphilis) into a Microsoft Excel worksheet (Microsoft Corporation, Redmond, Washington). Categorical variables were represented as percentages to calculate the prevalence of TTIs with respect to blood group and the total number of donations. The chi-square test was applied to compare the categorical variables and to identify any significant association. Odds ratios (ORs) for and against TTI infection, based on ABO and RhD blood groups, and their respective 95% confidence intervals (CIs) were estimated. Statistical analyses were performed at a 5% level of significance, with p < 0.05 considered statistically significant. Analyses were performed using IBM SPSS Statistics for Windows, Version 23 (Released 2015; IBM Corp., Armonk, New York).

## Results

A total of 65,160 whole blood donors were analyzed, of whom 98.2% (n = 63,987) were male and 1.8% (n = 1,173) were female; 95% (n = 61,902) were replacement donors and 5% (n = 3,258) were voluntary donors, indicating a markedly skewed donor profile dominated by males and heavily dependent on replacement donations, as shown in Table 1.

**Table 1:** Demographic details of the whole blood donor population. N: total number of whole blood donors.

Variable	Category	n (N = 65,160)	Percentage (%)
Gender	Male	63,987	98.2
	Female	1,173	1.8
Donor type	Replacement donors	61,902	95
	Voluntary donors	3,258	5
Donation status	First-time donors	63,467	97.4
	Repeat donors	1,693	2.6
Total donors	—	65,160	100

Prevalence of TTI

Table 2 summarizes the prevalence of TTIs according to ABO and RhD blood groups. Of the donors, 3.33% (n = 2,167) were seropositive for at least one marker. Among individual infections, HBV (n = 1017 (1.56%)) was the most prevalent, followed by syphilis (n = 655 (1.01%)), HCV (n = 268 (0.41%)), and HIV (n = 188 (0.29%)). Coinfections were found in 39 donors (0.06%), with the most frequent combinations being HBV with syphilis, HIV with HBV, and HCV with syphilis. Among RhD-positive donors, the highest number of TTI-positive donors was observed in blood group B-positive donors (n = 743 (3.30%)), followed by O-positive (n = 680 (3.34%)), A-positive (n = 464 (3.43%)), and AB-positive donors (n = 179 (3.31%)). However, among RhD-negative donors, the highest proportion of TTI seropositivity was found in AB-negative donors (3.74%), although the absolute number of cases was small due to the lower donation frequency.

Overall, the findings indicate that while RhD-positive donors constituted the majority of cases due to higher donation numbers, the relative prevalence of TTIs was slightly higher in RhD-negative groups, with AB-negative donors showing the highest proportional burden. HBV remained the predominant infection across all blood groups, followed by syphilis, HCV, and HIV, as shown in Table 2.

**Table 2:** Seroprevalence of TTI according to ABO and RhD blood groups. TTI: transfusion-transmitted infection; HIV: human immunodeficiency virus; HBV: hepatitis B virus; HCV: hepatitis C virus.

Blood group	Total number of donors	TTI-positive	HIV	HBV	HCV	Syphilis	Coinfection
A RhD-positive	13523 (20.75%)	464 (3.43%)	38 (0.28%)	229 (1.69%)	65 (0.48%)	123 (0.91%)	1 (HIV + HBV); 1 (HIV + HBV + HCV); 4 (HBV + Syphilis); 1 (HBV + HCV); 2 (HCV + Syphilis) (0.07%)
B RhD-positive	22498 (34.53%)	743 (3.30%)	63 (0.28%)	322 (1.43%)	96 (0.43%)	248 (1.10%)	3 (HIV + HBV); 3 (HIV + Syphilis); 4 (HBV + Syphilis); 2 (HBV + HCV); 2 (HCV + Syphilis) (0.06%)
O RhD-positive	20382 (31.28%)	680 (3.34%)	59 (0.29%)	336 (1.65%)	82 (0.40%)	189 (0.93%)	3 (HIV + HBV); 5 (HIV + Syphilis); 2 (HBV + HCV); 3 (HBV + Syphilis); 1 (HCV + Syphilis) (0.07%)
AB RhD-positive	5403 (8.29%)	179 (3.31%)	19 (0.35%)	88 (1.63%)	15 (0.28%)	56 (1.04%)	1 (HIV + HCV + Syphilis) (0.01%)
A RhD-negative	722 (1.11%)	18 (2.49%)	2 (0.28%)	8 (1.11%)	2 (0.28%)	6 (0.82%)	-
B RhD-negative	1224 (1.88%)	33 (2.69%)	2 (0.16%)	18 (1.48%)	2 (0.16%)	11 (0.89%)	-
O RhD-negative	1114 (1.71%)	39 (3.50%)	5 (0.45%)	13 (1.17%)	4 (0.35%)	17 (1.53%)	-
AB RhD-negative	294 (0.45%)	11 (3.74%)	0 (0%)	3 (1.02%)	2 (0.68%)	5 (1.70%)	1 (HIV + HCV) (0.34%)
Total	65160 (100%)	2167 (3.33%)	188 (0.29%)	1017 (1.56%)	268 (0.41%)	655 (1.01%)	39 (0.06%)

Association of ABO and RhD blood group with TTI positivity

Blood groups were compared with one another according to TTI prevalence using ORs (with 95% CIs), and p-values were calculated, as shown in Table 3.

**Table 3:** Association of ABO and RhD blood group with TTI. R: reactive, NR: nonreactive, OR: odds ratio, CI: confidence interval.

Blood group	HIV		OR (95%CI)	p-value, chi-square test	HBsAg		OR (95%CI)	p-value, chi-square test	HCV		OR (95%CI)	p-value	Syphilis		OR (95%CI)	p-value, chi-square test
	R	NR			R	NR			R	NR			R	NR		
A vs B, O, AB	40	14205	0.966	0.846	237	14008	1.087	0.262	67	14178	1.192	0.213	129	14116	0.875	0.177
	148	50767	(0.681-1.370)		780	50135	(0.939- 1.259)		201	50714	(0.904- 1.573)		526	50389	(0.721- 1.062)	
B vs A, O, AB	65	23657	0.923	0.601	340	23382	0.875	0.047	98	23624	1.007	0.956	259	23463	1.144	0.094
	123	41315	(0.683-1.247)		677	40761	(0.768- 0.998)		170	41268	(0.785- 1.292)		396	41042	(0.977- 1.339)	
O vs A, B, AB	64	21432	1.049	0.758	349	21147	1.062	0.364	86	21410	0.960	0.754	206	21290	0.931	0.400
	124	43540	(0.775- 1.418)		668	42996	(0.932- 1.210)		182	43482	(0.742- 1.241)		449	43215	(0.789- 1.099)	
AB vs A, B, O	19	5678	1.174	0.508	91	5606	1.026	0.816	17	5680	0.706	0.163	61	5636	1.073	0.604
	169	59294	(0.730- 1.888)		926	58537	(0.826- 1.275)		251	59212	(0.432- 1.155)		594	58869	(0.823- 1.398)	
RhD-positive vs RhD-negative	179	61627	1.080	0.823	975	60831	1.264	0.139	258	61548	1.402	0.293	616	61190	0.856	0.348

A statistically significant association was observed only between blood group B and HBV positivity (OR = 0.875, 95% CI: 0.768-0.998, p = 0.047). No significant associations were found between ABO or RhD blood groups and HIV, HCV, or syphilis. Overall, the distribution of TTIs did not show any consistent correlation with ABO or RhD blood group status.

Distribution and association of ABO and RhD blood groups and TTIs according to donor age

Donors were divided into three age groups, i.e., 18-30 years, 31-50 years, and >50 years. The distribution of ABO and RhD blood groups and TTI positivity was determined, as shown in Table 4.

Overall, the majority of reactive cases were concentrated in the younger than 50 years age group, with the least contribution from donors aged >50 years. This pattern was consistent across both ABO and RhD blood groups.

The association of donor age with TTIs is shown in Table 4. When comparing TTI reactivity, donors aged 18-30 years had significance lower (<0.001) than those aged 31-50 years, but significance higher than those aged >50 years. In contrast, donors aged 31-50 years had significantly higher odds of reactivity compared with other age groups. Donors aged >50 years showed the lowest prevalence.

**Table 4:** The association of donor age with transfusion-transmitted infections (TTIs). R: reactive, NR: nonreactive, OR: odds ratio, CI: confidence interval.

Age of donors (Years)	R (N = 2167)	NR (N = 62,993)	OR (N = 95%CI)	P
18-30 vs 31-50 (38,539) (20,520)	1174 (54.2%)	37,365 (59.31%)	0.672 (0.615- 0.734)	<0.001
	913 (42.1%)	19,607 (31.1%)		
18-30 vs >50 (38,539) (5927)	1174 (54.2%)	37,365 (59.31%)	2.286 (1.819- 2.872)	<0.001
	80 (3.7%)	5847 (9.3%)		
31-50 vs >50 (20,520) (5927)	913 (42.1%)	19,607 (31.1%)	3.403 (2.703- 4.285)	<0.001

## Discussion

The present study investigates the seroprevalence of TTIs among blood donors in Eastern India and explores their potential correlation with the ABO and RhD blood group system. Out of 65,160 donors screened, the overall TTI prevalence was 3.33%, with HBV being the most common infection (1.56%), followed by syphilis (1.01%), HCV (0.41%), and HIV (0.29%). Coinfections were uncommon (0.06%). These findings are higher than several previously reported estimates for the region and are consistent with data from other parts of India, where HBV continues to be the most frequently detected transfusion-transmissible infection among donors [13-15].

TTI positivity was relatively higher in Eastern India, largely due to lower awareness of safe, voluntary, non-remunerated blood donation and the continued reliance on replacement donors at greater than 90%, which allows individuals with undiagnosed infections to enter the donor pool [11]. Socioeconomic factors such as widespread poverty further limit access to timely healthcare, screening, and vaccination, particularly for hepatitis viruses, thereby increasing susceptibility to infection [2]. Additionally, substantial labor-related migration exposes individuals to diverse epidemiological environments, contributing to a higher overall burden of transfusion-transmissible infections [16].

Association with ABO and RhD blood groups

The overall seroprevalence of TTIs among all blood donors was 3.33%. No significant association was observed between the prevalence of TTIs and any specific ABO or RhD blood group, except for blood group B, which showed a significant association with HBV positivity [17]. A few Indian studies have highlighted links between ABO and RhD blood groups and TTIs. A study of 28,812 donors showed syphilis to be significantly more prevalent among blood group AB donors, with a p-value of 0.0331 [18]. Similarly, a study from North India of 36,614 blood donors observed higher HBsAg seropositivity among Rh-positive blood groups (p = 0.0459), while syphilis infection was significantly associated with blood group AB donors (p = 0.0331) [19]. However, our analysis did not reveal any overall statistically significant correlation between ABO or RhD blood groups and TTIs, except for a notable association between HBV seropositivity and blood group B (p = 0.047), suggesting that the relationship between ABO groups and infection susceptibility may vary across populations, as these associations are pathogen-specific and influenced by genetic and environmental factors. This must also be interpreted in the context of the ABO blood group frequency in our donor population, where blood group B predominates [20-21]. Overall, our study highlights that, at least in Eastern India, ABO grouping does not appear to be a strong predictor of overall TTI risk, though the association between HBV and blood group B warrants further exploration.

Age distribution and TTI risk

We observed that TTI seroreactivity was highest among donors aged 31-50 years, moderate among younger donors (18-30 years), and lowest in those older than 50 years, as shown in Table 5. This is consistent with earlier findings from different regions of India, where middle-aged groups often contribute significantly to donor pools and exhibit higher infection rates [18-19,22]. The lower prevalence in older donors could reflect survivor bias or reduced exposure risk. These findings underscore the importance of targeted awareness and preventive strategies in younger and middle-aged donor groups, who represent the bulk of the donor population. 

**Table 5:** Prevalence of seropositivity among different age groups.

Age of donors (years)	Reactive	Non-reactive
18-30 (38,539)	1174 (3.04%)	37,365 (96.95%)
31-50 (20,520)	913 (4.44%)	19,607 (95.55%)
>50 (5927)	80 (1.34%)	5847 (98.65%)

Implications for blood safety

Despite advances in serological testing (enzyme-linked immunosorbent assay (ELISA)/chemiluminescent immunoassay (CLIA)) and optional nucleic acid testing (NAT), the persistence of TTIs among donors continues to pose challenges to transfusion safety. Our findings reinforce the need for continuous monitoring, enhanced public health education, and implementation of stringent donor selection criteria. Regional variations in prevalence, particularly the relatively higher burden of syphilis and the significant association between HBV and blood group B donors, suggest that donor epidemiology should guide transfusion policies at the local level.

Limitations

This study has several limitations. First, it was conducted at a single blood center in Eastern India, limiting the generalizability of the findings to other populations and geographical regions with different donor characteristics and epidemiological profiles. Second, the study population was predominantly male (98.2%) and comprised replacement donors (95%), with limited representation of female and voluntary donors, potentially introducing selection bias and reducing applicability to these groups. Third, the cross-sectional design permits estimation of TTI seroprevalence but cannot establish temporal or causal relationships between ABO/RhD blood groups, age, and TTI positivity.

## Conclusions

Our study demonstrates that HBV remains the leading TTI among donors in Eastern India, with a statistically significant association between HBsAg positivity and blood group B. Although no overall correlation between ABO/RhD blood groups and TTIs was established, these findings highlight the continued public health burden of HBV and the importance of region-specific donor screening strategies. Sustained awareness programs, rigorous donor selection, and the incorporation of advanced testing methods are crucial to further enhancing blood safety in India.
